# Supplementary material for: Predicted structure of fully activated human bitter taste receptor TAS2R4 complexed with G protein and agonists
Source: QRB Discov. 2021 Apr 8;2:e3. doi: 10.1017/qrd.2021.1 (PMC10392674; doi:10.1017/qrd.2021.1)
Supplement: Supplementary file 1 [file S2633289221000016sup001.docx]

**Supporting Information**

Predicted Structure of Fully Activated Human Bitter Taste Receptor TAS2R4 Complexed with G Protein and Agonists

**Moon Young Yang^a^, Amirhossein Mafi^a^, Soo-Kyung Kim^a^, William A. Goddard III^a,1^, and Brian Guthrie^b^**

^a^Materials and Process Simulation Center, California Institute of Technology, Pasadena, CA, 91125

^b^Cargill Global Food Research, Wayzata, MN 55391

^1^**Corresponding author:** wag@caltech.edu

This document includes**:**

Figures S1 to S16

Tables S1 and S8

SI References

**Table S1.** Sequence identity of TAS2R4 against Class A GPCRs for which active structures are available. We elected the four templates (bold) for the GEnSeMBLE optimization. Only the templates AT_2_ and 5-HT_2C_ were represented in the top 10 for the BiHelix step. Our final structure was based on 5-HT_2C_.

|  | TMD (%) | | All sequence (%) | |
| --- | --- | --- | --- | --- |
|  | Seq^a^ | Seq^b^ | Seq^a^ | Seq^b^ |
| Adenosine A_1_ | 9.87 | 7.53 | 8.93 | 6.84 |
| Adenosine A_2A_ | 10.51 | 9.05 | 6.4 | 6.56 |
| **Angiotensin_2_** | **14.36** | **13.11** | **7.61** | **8.06** |
| β_2_ adrenergic | 11.02 | 10.25 | 6.62 | 6.85 |
| **Cannabinoid_1_** | **9.53** | **12.45** | **4.9** | **6.94** |
| 5-HT_1B_ | 12.44 | 11.3 | 9.34 | 7.23 |
| 5-HT_2B_ | 11.73 | 10.7 | 5.05 | 5.93 |
| **5-HT_2C_** | **13.33** | **13.52** | **6.33** | **7.42** |
| κ opioid | 9.88 | 11.3 | 6.58 | 7.16 |
| M2 muscarinic | 11.74 | 11.43 | 6.68 | 6.72 |
| **Rhodopsin** | **13.35** | **11.49** | **8.26** | **8.89** |
| μ opioid | 11.53 | 10.04 | 6.73 | 6.6 |
| Neurotensin_1_ | 13.55 | 9.96 | 7.69 | 7 |

Sequence alignment was carried out based on database ^a^ref.1 and ^b^ref.2, respectively.

**Table S2**. The top 10 structures from the BiHelix calculation for predicting the TMD of TAS2R4. These were selected from the 4000 7-helix bundles for the 4 templates: serotonin 2C (5-HT_2C_), angiotensin 2 (AT_2_), cannabinoid 1 (CB_1_), and rhodopsin. They are ordered by neutral interhelical energy (Nih). We selected the 1st (from AT_2_) and 3rd (from 5-HT_2_c) structures (bold) for the SuperBiHelix step. Only the templates AT_2_ and 5-HT_2C_ were represented in the top 10.

| # | Eta | | | | | | | rankCih | rankNih | rankCNti | Source |
| --- | --- | --- | --- | --- | --- | --- | --- | --- | --- | --- | --- |
|  | H1 | H2 | H3 | H4 | H5 | H6 | H7 |  |  |  |  |
| **1** | **0** | **0** | **0** | **0** | **-120** | **0** | **0** | **206** | **1** | **37** | **AT_2_** |
| 2 | 0 | 0 | 0 | 0 | 180 | 0 | 0 | 363 | 2 | 44 | AT_2_ |
| **3** | **0** | **0** | **0** | **90** | **0** | **0** | **0** | **1301** | **3** | **318** | **5-HT_2C_** |
| 4 | 0 | 0 | 0 | 0 | -150 | 0 | 0 | 1072 | 4 | 245 | 5-HT_2C_ |
| 5 | 0 | 0 | 0 | 180 | -90 | 0 | 0 | 666 | 5 | 132 | 5-HT_2C_ |
| 6 | 0 | 0 | 0 | 30 | 180 | 0 | 0 | 475 | 6 | 89 | AT_2_ |
| 7 | 0 | 0 | 0 | -30 | -120 | 0 | 0 | 516 | 7 | 151 | AT_2_ |
| 8 | 0 | 0 | 0 | 0 | 150 | 0 | 0 | 1714 | 8 | 533 | AT_2_ |
| 9 | 0 | 0 | 0 | 150 | -30 | 0 | 0 | 157 | 9 | 50 | AT_2_ |
| 10 | 0 | 0 | 0 | 180 | -30 | 0 | 0 | 1269 | 10 | 391 | 5-HT_2C_ |

**Table S3**. The top 25 structures after the SuperBiHelix calculations for the 2 cases selected from BiHelix. These are selected from the 2000 top structures from SuperBiHelix. They are ordered by total energy (CNti: the average of total and interhelical energy of charged and neutral). We selected the 1st structure (bold) for further study.

| # | Theta | | | | | | | Phi | | | | | | | Eta | | | | | | | rankCih | rankNih | rankCNti | Source |
| --- | --- | --- | --- | --- | --- | --- | --- | --- | --- | --- | --- | --- | --- | --- | --- | --- | --- | --- | --- | --- | --- | --- | --- | --- | --- |
|  | H1 | H2 | H3 | H4 | H5 | H6 | H7 | H1 | H2 | H3 | H4 | H5 | H6 | H7 | H1 | H2 | H3 | H4 | H5 | H6 | H7 |  |  |  |  |
| **1** | **0** | **0** | **0** | **10** | **0** | **0** | **0** | **-15** | **0** | **0** | **-15** | **15** | **15** | **0** | **0** | **0** | **0** | **30** | **0** | **0** | **0** | **10** | **2** | **1** | **5-HT_2C_** |
| 2 | -10 | -10 | 0 | -10 | 10 | 0 | -10 | -15 | 0 | 15 | 30 | 0 | 0 | -15 | 15 | 0 | 0 | 30 | -30 | 15 | -30 | 42 | 8 | 2 | AT_2_ |
| 3 | 0 | 0 | 0 | 10 | 0 | 0 | 0 | -15 | 0 | 0 | 0 | 15 | 15 | 0 | 0 | 0 | 0 | 0 | 0 | 0 | 0 | 75 | 21 | 3 | 5-HT_2C_ |
| 4 | -10 | -10 | 0 | -10 | 10 | 0 | -10 | -15 | 0 | 15 | 30 | 0 | -15 | -15 | 15 | 0 | 0 | 30 | 15 | 15 | -30 | 97 | 11 | 4 | AT_2_ |
| 5 | -10 | -10 | 0 | 0 | 0 | 0 | -10 | -15 | 0 | 0 | -30 | 0 | -15 | -15 | 0 | 0 | 15 | 15 | 0 | 0 | 15 | 77 | 28 | 5 | 5-HT_2C_ |
| 6 | -10 | -10 | 0 | 0 | 0 | 0 | -10 | -15 | 0 | 0 | -30 | 0 | -30 | 0 | 15 | 0 | 15 | 15 | 0 | 0 | 15 | 116 | 19 | 6 | 5-HT_2C_ |
| 7 | -10 | -10 | 0 | 0 | 0 | 0 | -10 | 0 | 0 | 0 | -30 | 0 | -15 | -15 | 15 | 0 | 15 | 15 | 0 | 0 | 15 | 82 | 15 | 7 | 5-HT_2C_ |
| 8 | 0 | -10 | 0 | 0 | 0 | 0 | -10 | -15 | 0 | 0 | -30 | 0 | -15 | -15 | -15 | 0 | 15 | 15 | 0 | 0 | 15 | 83 | 13 | 8 | 5-HT_2C_ |
| 9 | 0 | 0 | 0 | 10 | 0 | 0 | 0 | 0 | 0 | 0 | -15 | 15 | 15 | 0 | 0 | 0 | 0 | 15 | -30 | 0 | 0 | 1 | 1 | 9 | 5-HT_2C_ |
| 10 | 0 | -10 | 0 | -10 | 10 | 0 | -10 | -15 | 0 | 15 | 30 | 0 | -15 | 0 | 0 | 0 | 0 | 30 | -30 | 15 | 0 | 58 | 56 | 10 | AT_2_ |
| 11 | 0 | 0 | 0 | 10 | 0 | 0 | 0 | -15 | 0 | 0 | -15 | 15 | 15 | 0 | 0 | 0 | 0 | 30 | -15 | 0 | 0 | 13 | 18 | 11 | 5-HT_2C_ |
| 12 | -10 | -10 | -10 | 0 | 0 | -10 | -10 | 0 | -15 | 0 | -15 | -30 | -15 | 15 | 15 | 15 | 0 | 0 | -15 | 15 | -30 | 9 | 26 | 12 | AT_2_ |
| 13 | 0 | -10 | 0 | -10 | 10 | 0 | -10 | -15 | 0 | 15 | 15 | 0 | -15 | 0 | 15 | 0 | 0 | 30 | -30 | 15 | 0 | 73 | 148 | 13 | AT_2_ |
| 14 | -10 | -10 | 0 | 0 | 0 | 0 | -10 | 0 | 0 | 0 | -30 | 0 | -15 | -15 | 15 | 0 | 15 | 15 | -15 | 0 | 15 | 217 | 27 | 14 | 5-HT_2C_ |
| 15 | -10 | -10 | 0 | -10 | 10 | 0 | -10 | 0 | 0 | 15 | 30 | 0 | -15 | 0 | 15 | 0 | 0 | 30 | -30 | 15 | 0 | 72 | 61 | 15 | AT_2_ |
| 16 | -10 | -10 | 0 | -10 | 10 | 0 | -10 | -15 | 0 | 15 | 30 | -15 | -15 | 15 | 15 | 0 | 0 | 30 | 0 | 15 | -30 | 201 | 55 | 16 | AT_2_ |
| 17 | 0 | 0 | 0 | 10 | 0 | 0 | 0 | 0 | 0 | 0 | -30 | -15 | 15 | 0 | 0 | 0 | 0 | -30 | 15 | 0 | 0 | 69 | 40 | 17 | 5-HT_2C_ |
| 18 | -10 | -10 | 0 | -10 | 10 | 0 | -10 | -15 | 0 | 15 | 30 | 0 | 0 | 0 | 15 | 0 | 0 | 30 | -30 | 15 | -15 | 32 | 83 | 18 | AT_2_ |
| 19 | -10 | -10 | 0 | 0 | 0 | 0 | -10 | 0 | 0 | 0 | -30 | 0 | 30 | 15 | 15 | 0 | 15 | 15 | 0 | 15 | -15 | 7 | 3 | 19 | 5-HT_2C_ |
| 20 | 0 | 0 | 0 | 10 | 0 | 0 | 0 | -15 | 0 | 0 | 0 | 15 | 15 | 0 | -15 | 0 | 0 | 0 | 0 | 0 | 0 | 294 | 80 | 20 | 5-HT_2C_ |
| 21 | 0 | -10 | 0 | -10 | 10 | -10 | -10 | -15 | 0 | 0 | 30 | -15 | 30 | 30 | 0 | 0 | -15 | 30 | -30 | 15 | -15 | 22 | 85 | 21 | AT_2_ |
| 22 | -10 | -10 | 0 | -10 | 10 | 0 | -10 | -15 | 0 | 15 | 30 | 0 | -15 | 0 | 15 | 0 | 0 | 30 | -30 | 15 | 0 | 246 | 99 | 22 | AT_2_ |
| 23 | -10 | -10 | 0 | -10 | 10 | 0 | -10 | 0 | 0 | 15 | 15 | 0 | -15 | 0 | 15 | 0 | 0 | 30 | 15 | 15 | 0 | 43 | 89 | 23 | AT_2_ |
| 24 | 0 | -10 | 0 | -10 | 10 | -10 | -10 | -15 | 0 | 0 | 30 | -30 | -15 | 15 | 0 | 0 | -15 | 30 | 0 | 15 | -15 | 104 | 163 | 24 | AT_2_ |
| 25 | -10 | -10 | 0 | 0 | 0 | 0 | -10 | -15 | 0 | 0 | -30 | 0 | -15 | -15 | 30 | 0 | 15 | 15 | 0 | 0 | 15 | 164 | 25 | 25 | 5-HT_2C_ |


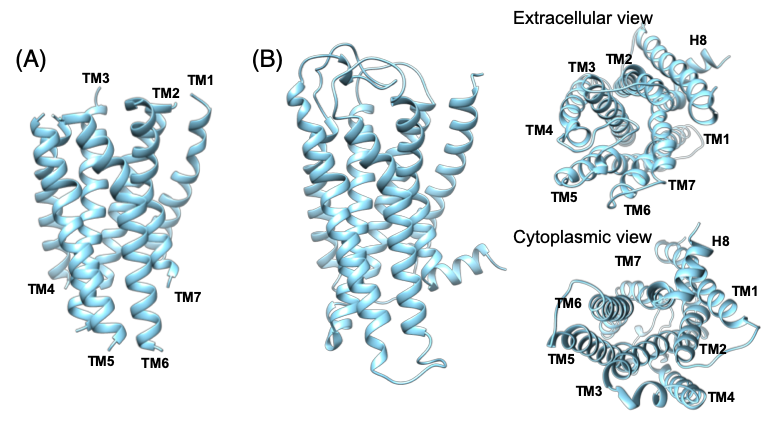


**Fig. S1.** (A) The predicted TMD structure of TAS2R4 predicted from GEnSeMBLE and (B) the structure after attaching loops.

**Table S4**. Agonists for TAS2R4 that exhibit binding equal or lower than 100 μM effective concentration.^3^ We studied the two in bold face.

| Ligand | Specificity to TAS2Rs | Effective concentration (μM) |
| --- | --- | --- |
|  |  |  |
| **quinine** | 4, 7, 10, 14, 39, 40, 43, 44, 46 | 10 |
| Trp-Trp-Trp | 1, 4, 14, 39, 46 | 10 |
| arborescin | 1, 4, 10, 14, 43, 46 | 30 |
| parthenolide | 1, 4, 8, 10, 14, 44, 46 | 30 |
| chlorpheniramine | 4, 7, 10, 14, 38, 39, 40, 46 | 30 |
| **rubusoside** | 4, 14 | 50 |
| artemorin | 4, 10, 14, 46, 47 | 100 |
| diphenidol | 1, 4, 7, 10, 13, 14, 16, 38,  39, 40, 43, 44, 46, 47, 49 | 100 |
| colchicine | 4, 39, 46 | 100 |
| dapsone | 4, 10, 40 | 100 |


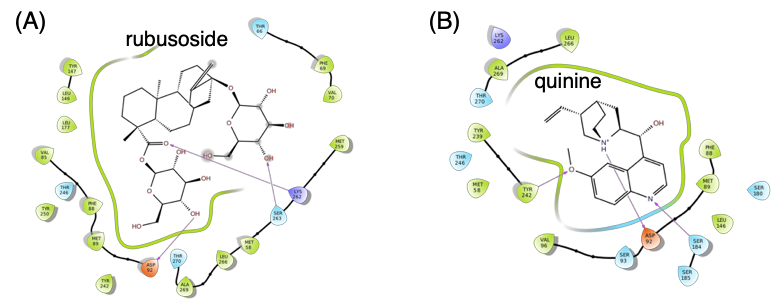


**Fig. S2.** Pharmacophores for the DarwinDock predicted structures to the TMD of TAS2R4, (A) rubusoside and (B) quinine.

**
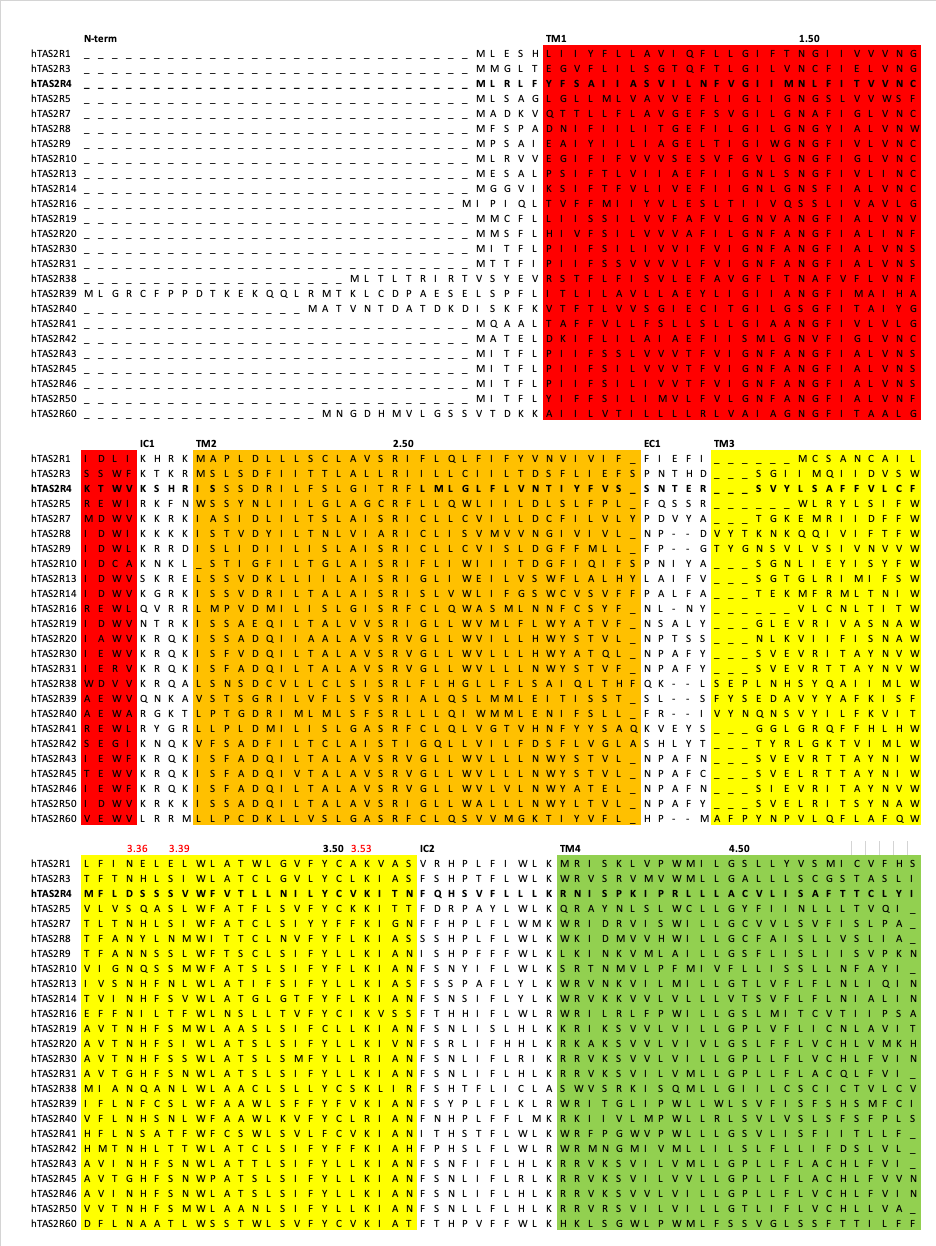
**

**
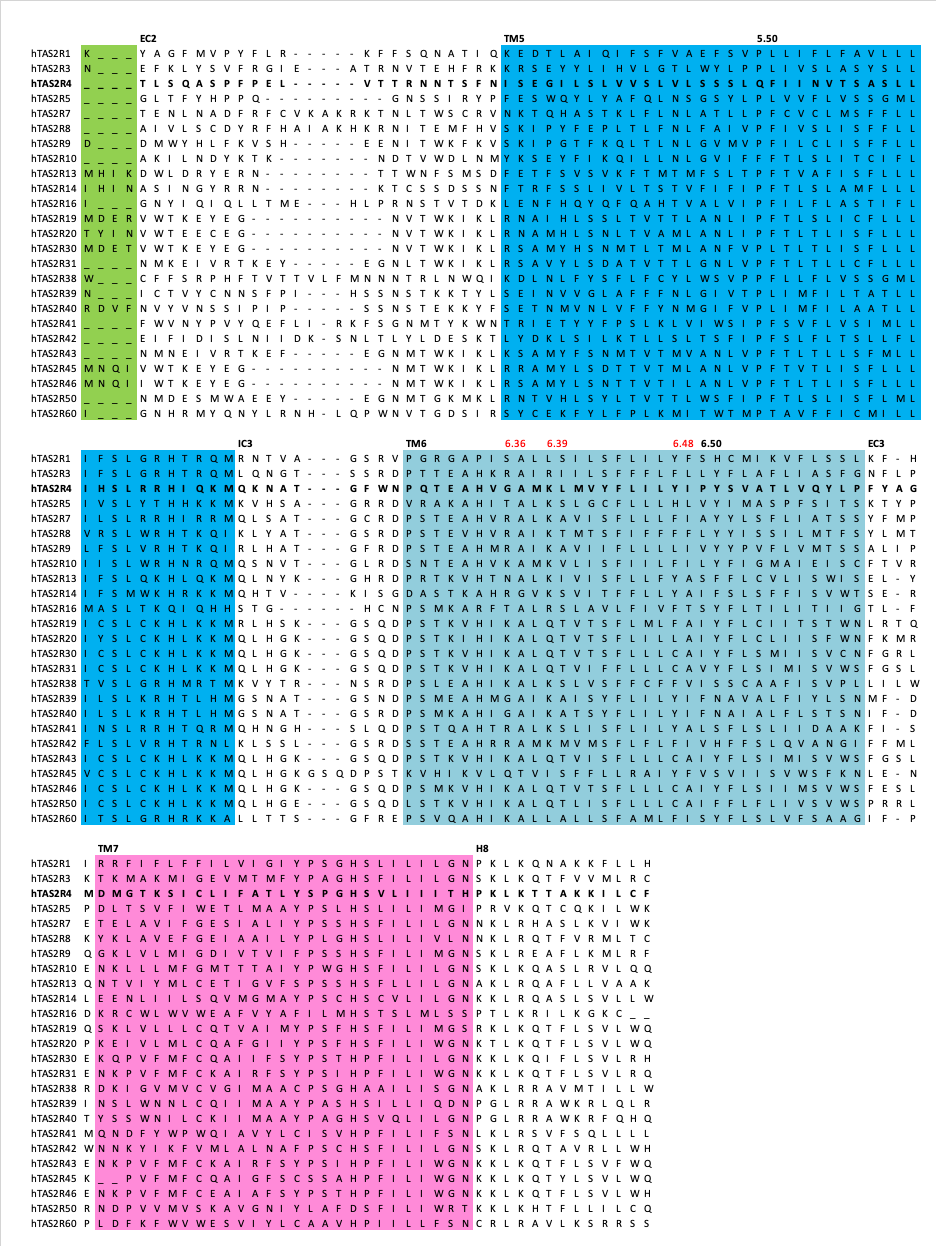
**

**Fig. S3.** Sequence alignment of 25 bitter taste receptors (TAS2Rs).^2^

**Table S5.** The structure of steviol glycosides and the obtained experimental bitterness data at 300 ppm.

**
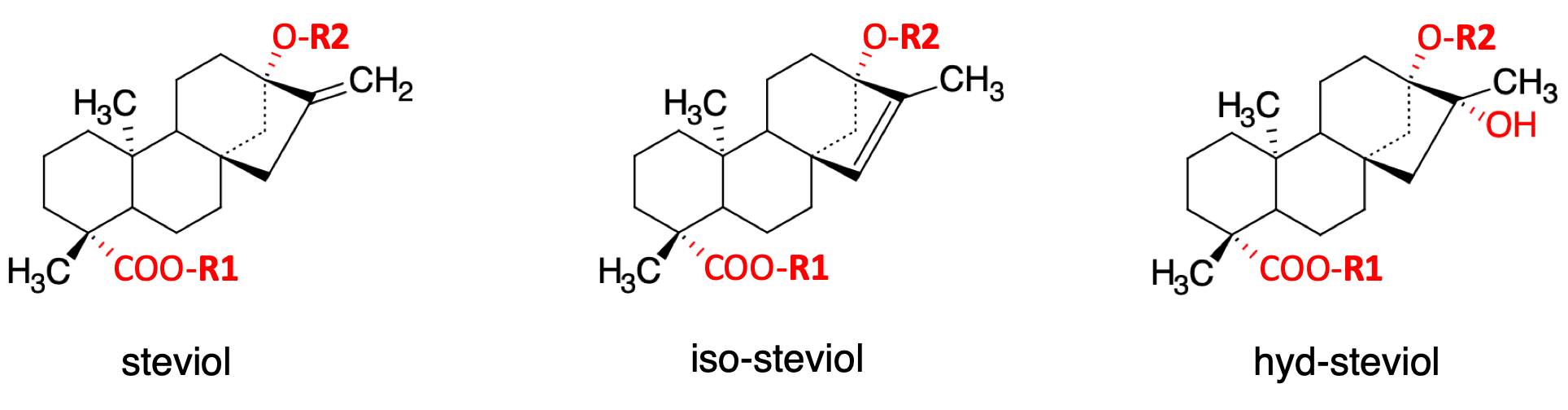
**

| **Steviol glycosides** | **R1 (COO-)** | **R2 (O-)** | **Bitterness (300 ppm)** |
| --- | --- | --- | --- |
| Rubu | Gluβ1- | Gluβ1- | 4.04 |
| stevioside | Gluβ1- | Gluβ1-2Gluβ1- | 3.41 |
| RebG | Gluβ1- | Gluβ1-3Gluβ1- | 3.02 |
| RebA | Gluβ1- | Gluβ1-2(Gluβ1-3)Gluβ1- | 2.97 |
| RebD | Gluβ1-2Gluβ1- | Gluβ1-2(Gluβ1-3)Gluβ1- | 2.79 |
| isoRebM | Gluβ1-2(Gluβ1-3)Gluβ1- | Gluβ1-2(Gluβ1-3)Gluβ1- | 2.68 |
| RebM | Gluβ1-2(Gluβ1-3)Gluβ1- | Gluβ1-2(Gluβ1-3)Gluβ1- | 2.41 |
| RebN | Rhaα1-2(Gluβ1-3)Gluβ1- | Gluβ1-2(Gluβ1-3)Gluβ1- | 2.33 |
| RebB | - | Gluβ1-2(Gluβ1-3)Gluβ1- | 2.27 |
| RebO | Gluβ1-3Rhaα1-2(Gluβ1-3)Gluβ1- | Gluβ1-2(Gluβ1-3)Gluβ1- | 2.24 |
| RebE | Gluβ1-2Gluβ1- | Gluβ1-2Gluβ1- | 2.14 |
| RebC | Gluβ1- | Rhaα1-2(Gluβ1-3)Gluβ1- | 1.72 |
| hydRebM | Gluβ1-2(Gluβ1-3)Gluβ1- | Gluβ1-2(Gluβ1-3)Gluβ1- | 1.07 |

- Reb: Rebaudioside, Gluβ: beta glucose, Rhaα: alpha rhamnose

**Table S6.** Average interaction energies (kJ/mol) (Coulomb (Coul), van der Waals (vdW), and total energies, respectively) between ligands and TAS2R4 for 480 ns MD simulations.

| **Residue** | **Rubu** | | | **Quinine** | | | **RebM** | | | **hydRebM** | | |
| --- | --- | --- | --- | --- | --- | --- | --- | --- | --- | --- | --- | --- |
|  | Coul | vdW | Total | Coul | vdW | Total | Coul | vdW | Total | Coul | vdW | Total |
| D92^3.36^ | -112.8 | 10.2 | -102.6 | -84.5 | 1.4 | -83.1 | -115.8 | 8.2 | -107.6 | -140.5 | -1.3 | -141.8 |
| M89^3.33^ | -6.2 | -24.7 | -30.9 | -0.3 | -8.1 | -8.4 | -2.5 | -14.8 | -17.4 | -5.5 | -19.2 | -24.8 |
| Y250^6.59^ | 0.1 | -27.0 | -27.0 | -0.9 | -4.0 | -5.0 | -5.7 | -10.9 | -16.6 | -4.4 | -17.7 | -22.1 |
| Y147^4.62^ | -4.6 | -21.0 | -25.5 |  |  |  | -22.9 | -20.2 | -43.1 | -5.0 | -19.5 | -24.5 |
| L177^5.39^ | -3.6 | -20.7 | -24.3 | -0.1 | -3.0 | -3.1 | 0.1 | -9.8 | -9.7 | 0.0 | -4.1 | -4.1 |
| S93^3.37^ | -19.3 | -3.2 | -22.5 | -2.5 | 0.3 | -2.2 | -0.2 | -1.9 | -2.0 | -15.8 | -5.4 | -21.1 |
| L181^5.43^ | 0.5 | -21.1 | -20.6 | -1.7 | -12.7 | -14.5 | 0.3 | -12.0 | -11.8 | -0.9 | -18.2 | -19.1 |
| E158^ECL2^ | -17.1 | -1.6 | -18.6 |  |  |  | -26.1 | -23.2 | -49.3 | -24.4 | -9.3 | -33.7 |
| N164^ECL2^ | -9.9 | -8.4 | -18.3 |  |  |  | 1.8 | -8.9 | -7.2 |  |  |  |
| T162^ECL2^ | -3.2 | -7.8 | -10.9 |  |  |  | -1.9 | -14.9 | -16.8 | -8.2 | -12.2 | -20.5 |
| F88^3.32^ | -2.4 | -7.7 | -10.1 | -0.5 | -5.0 | -5.5 | -2.5 | -22.1 | -24.6 | -1.1 | -12.6 | -13.7 |
| Q188^5.50^ | -1.0 | -0.8 | -1.7 | -6.3 | -3.0 | -9.3 |  |  |  | -5.1 | -3.7 | -8.8 |
| Y242^6.51^ | 0.6 | -1.7 | -1.1 | -3.9 | -11.2 | -15.1 | -4.3 | -5.7 | -10.0 | -29.8 | -19.5 | -49.3 |
| N65^2.60^ |  |  |  |  |  |  | -25.3 | -11.4 | -36.7 | -6.3 | -6.1 | -12.5 |
| Y68^2.63^ |  |  |  |  |  |  | 0.5 | -14.2 | -13.7 | -24.9 | -13.5 | -38.4 |
| F69^2.64^ |  |  |  |  |  |  | -13.4 | -26.1 | -39.5 | -2.0 | -12.7 | -14.7 |
| S72^ECL1^ |  |  |  |  |  |  | -33.2 | -5.8 | -39.0 | -2.8 | -8.1 | -10.9 |
| E75^ECL1^ |  |  |  |  |  |  | -58.6 | -0.3 | -58.8 | -2.0 | -1.3 | -3.3 |
| D258^7.31^ |  |  |  |  |  |  | -90.7 | 2.9 | -87.8 | -117.1 | 10.0 | -107.1 |
| K262^7.35^ |  |  |  |  |  |  | -16.5 | -17.3 | -33.8 | -14.0 | -11.7 | -25.6 |
| Total | -189.5 | -244.8 | -434.4 | -98.2 | -90.5 | -188.7 | -461.9 | -360.0 | -822.0 | -425.9 | -341.5 | -767.7 |


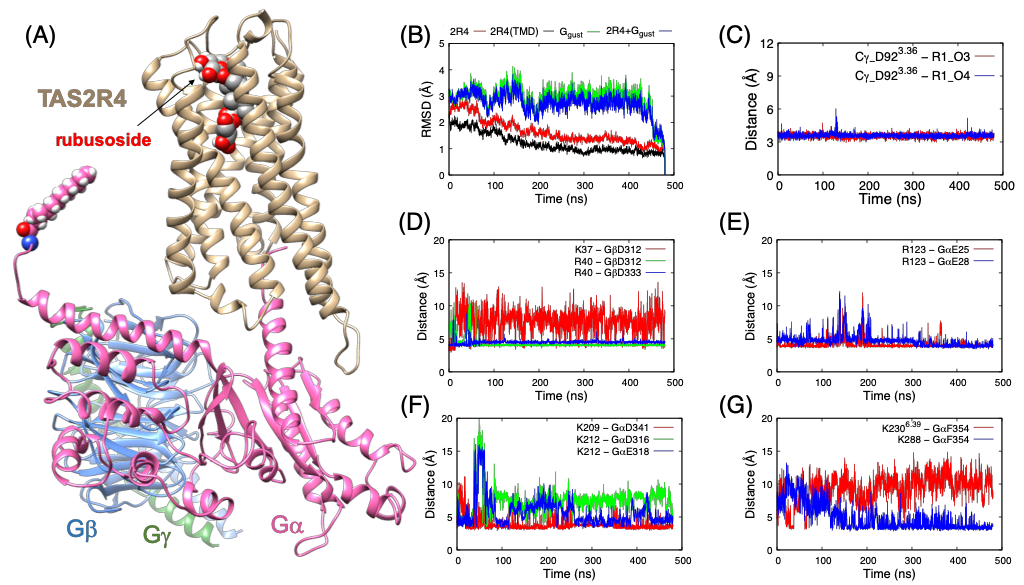


**Fig. S4.** The TAS2R4-Rubu-G_gust_ structure and results of 480 ns MD simulation. (A) The equilibrated complex structure. (B) RMSD plots for backbone atoms of TAS2R4 (red), TMD of TAS2R4 (black), G_gust_ (green) , and the full complex (blue), and (C) time evolution for the distance between D92^3.36^ and the oxygen atoms in sugar ring of Rubu. (D) Distance between residues for forming SBs in anchor 1, (E) same for anchor 2, and (F) same for anchor 3. (F) Distance between the terminal carboxylate of F354Gα5 and K230^6.39^ or K288H8.


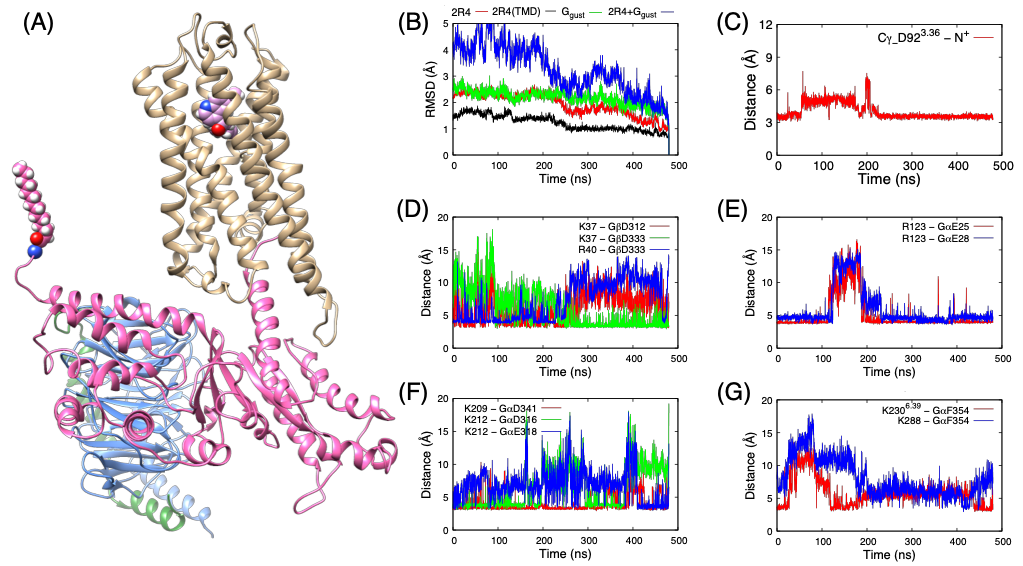


**Fig. S5.** The TAS2R4-quinine-G_gust_ structure and results of 480 ns MD simulation. (A) The equilibrated complex structure. (B) RMSD plots for backbone atoms of TAS2R4 (red), TMD of TAS2R4 (black), G_gust_ (green) , and the full complex (blue), respectively. (C) Distance between D92^3.36^ and the tertiary nitrogen of quinine. Distances between residues for forming SBs in (D) anchor 1, (E) anchor 2, and (F) anchor 3, respectively. (G) Distance between the terminal carboxylate of F354Gα5 and K230^6.39^ or K288H8.

**
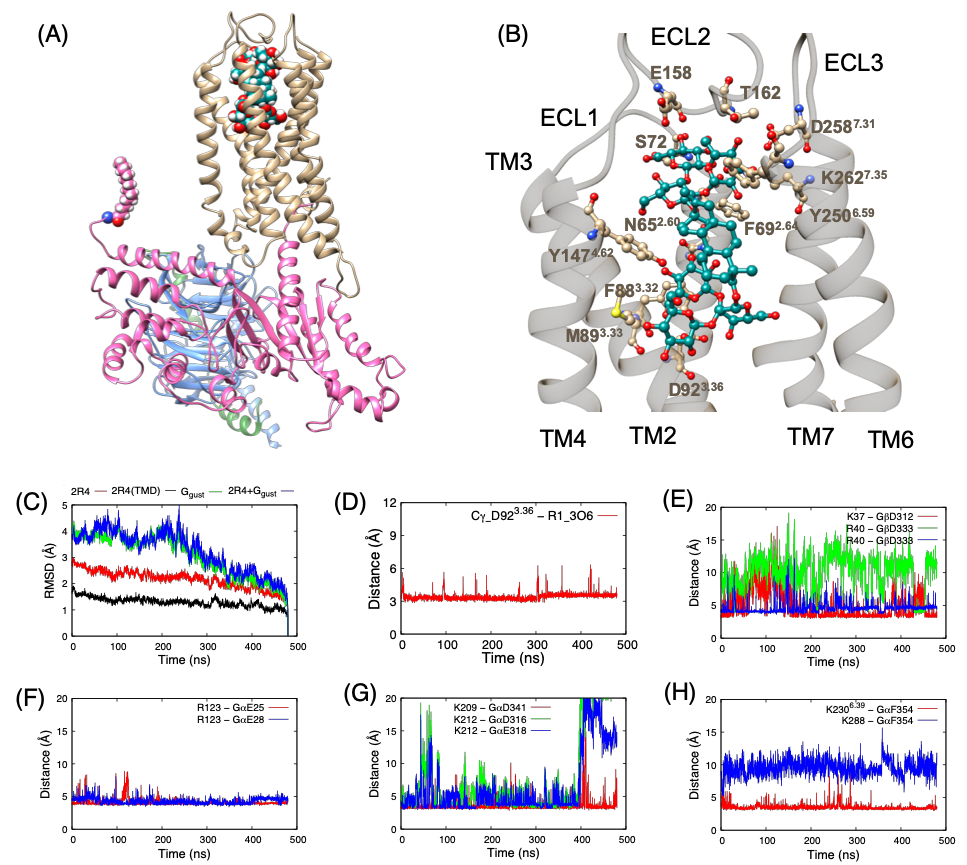
**

**Fig. S6.** (A) The TAS2R4-RebM-G_gust_ structure and (B) its binding site. (C) RMSD plots for backbone atoms of TAS2R4 (red), TMD of TAS2R4 (black), G_gust_ (green) , and the full complex (blue), and (D) distance between D92^3.36^ and the oxygen in the sugar ring of RebM that makes a HB. Distances between residues for forming SBs in (E) anchor 1, (F) anchor 2, and (G) anchor 3, respectively, for 480 ns MD simulation. (H) Distance between the terminal carboxylate of F354Gα5 and K230^6.39^ or K288H8.

**
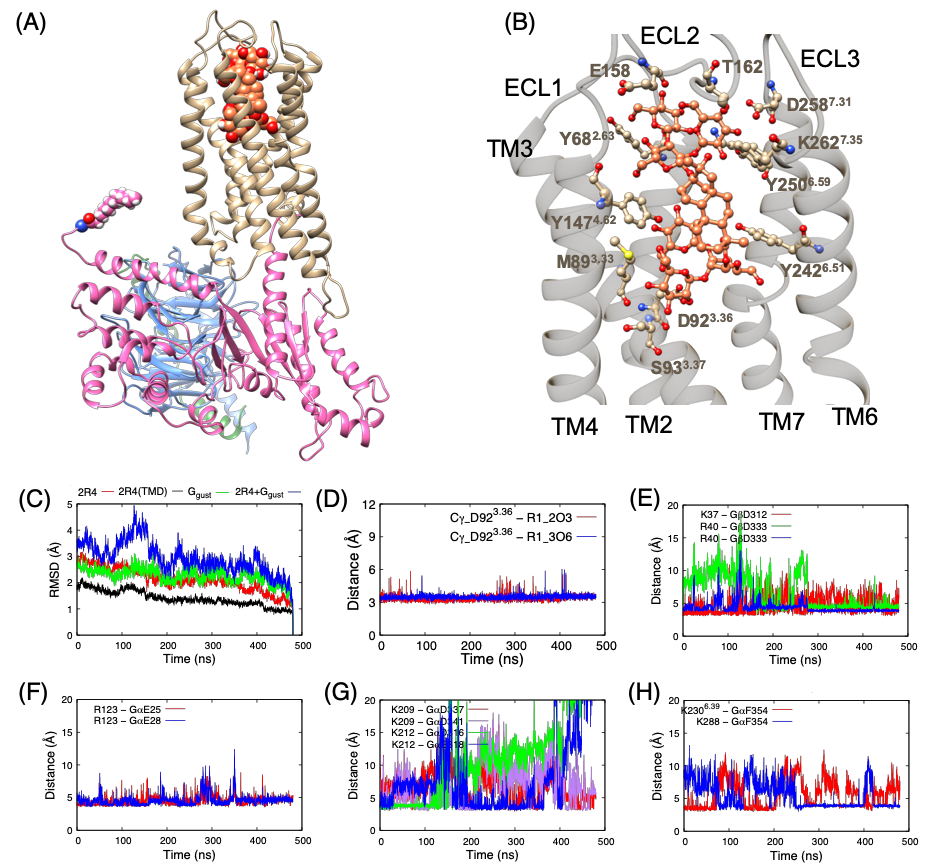
**

**Fig. S7.** (A) The TAS2R4-hydRebM-G_gust_ structure and (B) its binding site. (C) RMSD plots for backbone atoms of TAS2R4 (red), TMD of TAS2R4 (black), G_gust_ (green) , and the full complex (blue), and (D) distances between D92^3.36^ and the oxygens in the sugar ring of hydRebM that makes HBs. Distances between residues for forming SBs in (E) anchor 1, (F) anchor 2, and (G) anchor 3, respectively, for 480 ns MD simulation. (H) Distance between the terminal carboxylate of F354Gα5 and K230^6.39^ or K288H8.

**
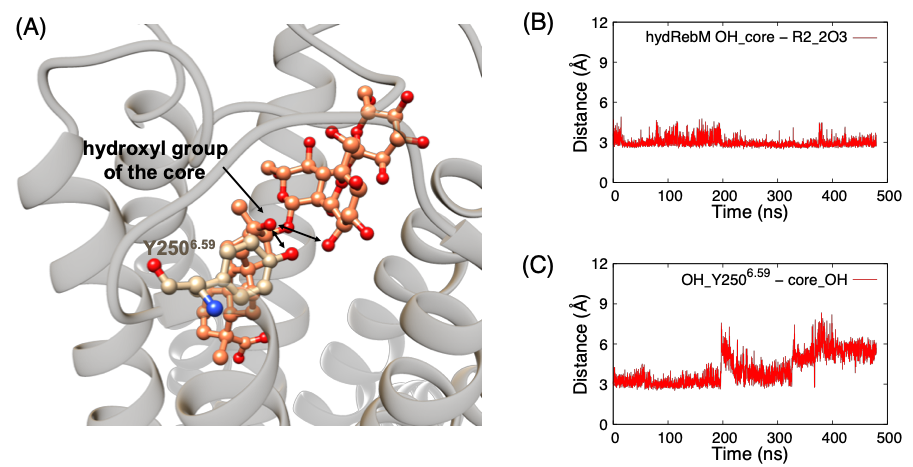
**

**Fig. S8.** Interactions of the hydroxyl group of the hydRebM core structure.

**
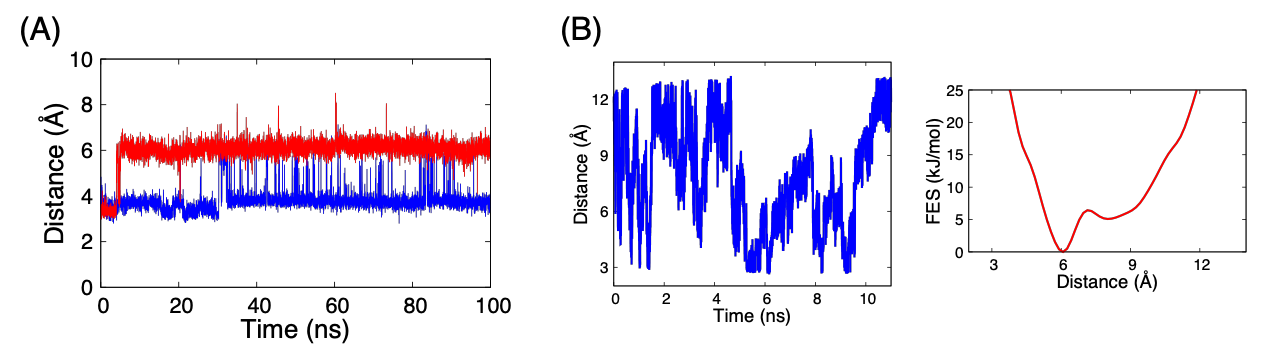
**

**Fig. S9.** (A) Distance between D92^3.36^ and Y239^6.48^ in the agonist absent model 1 (blue) and model 2 (red), respectively. Distances of ~4 Å and ~6 Å correspond to the direct and water-mediated HBs, respectively. (B) Free energy analysis by metaMD for interactions between D92^3.36^ – Y239^6.48^, where variations of interactions with time (left) and the estimated free energy profile (right) are shown.

**
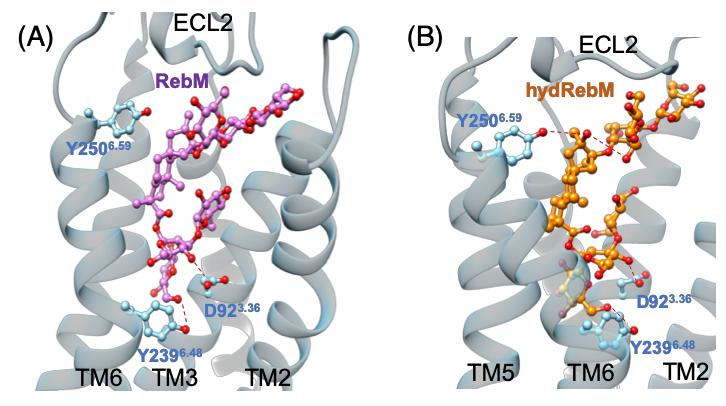
**

**Fig. S10.** Predicted structures of (A) RebM and (B) hydRebM bound to the pre-activated state of TAS2R4.


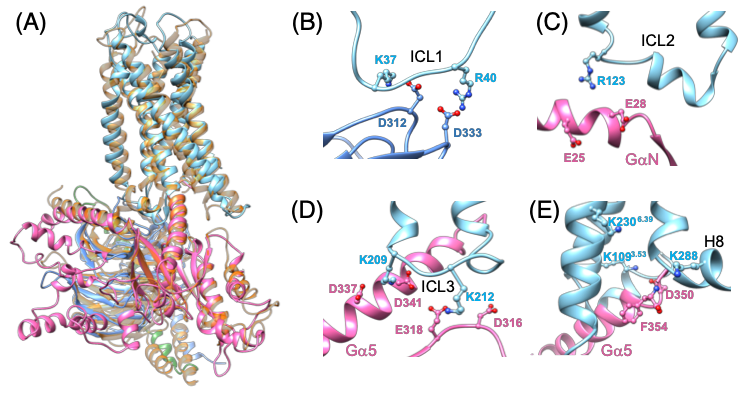


**Fig. S11.** (A) The initial structures of predicted TAS2R4 (cyan) and G_gust_ (magenta, blue, and green) superimposed on the TMD and G_i_P of the mouse μOR-G_i_ crystal structure (orange; PDBID: 6DDF), respectively. Charged residues for (B-D) the anchor formation and (E) the Gα5 helix positioning between GPCR and G_gust_.

**
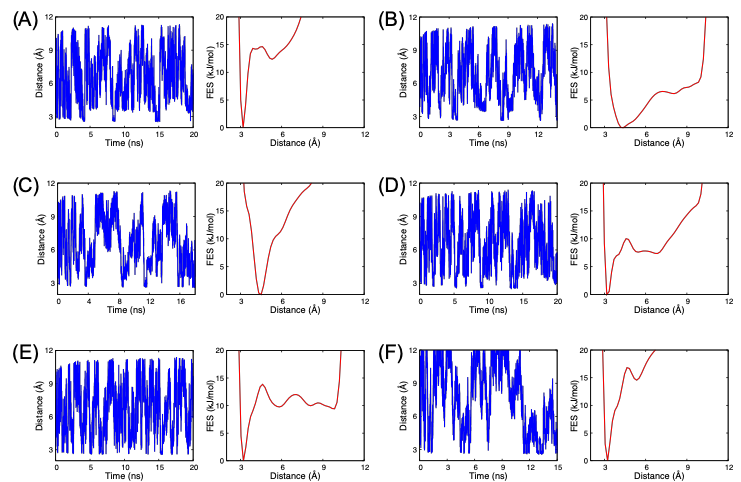
**

**Fig. S12.** Free energy analysis by metaMD for interactions between TAS2R4 and G_gust_, (A) K37ICL1 - D312Gβ, (B) R40ICL1 - D333Gβ, (C) R123ICL2 – E28GαN, (D) K209ICL3 - D341Gα5, (E) K212ICL3 – E318Gα, (F) K230^6.39^ – F354Gα5, respectively.


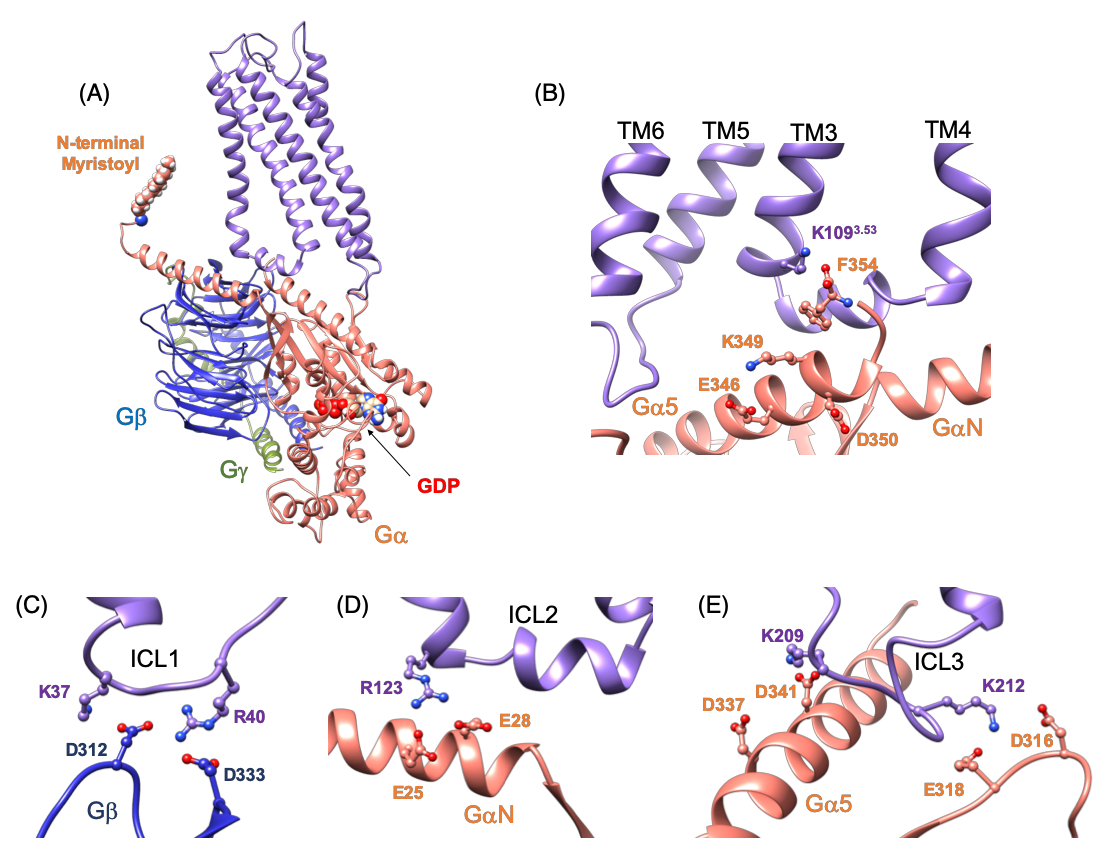


**Fig. S13.** (A) Predicted structure of the pre-activated state of TAS2R4-G_gust_ complex, where inactive G_gust_ is tightly bound to GDP. (B) The terminal carboxylate of F354Gα5 makes a SB with K109^3.53^. Polar interactions in (C) anchor 1, (D) anchor 2, and (E) anchor 3, respectively.


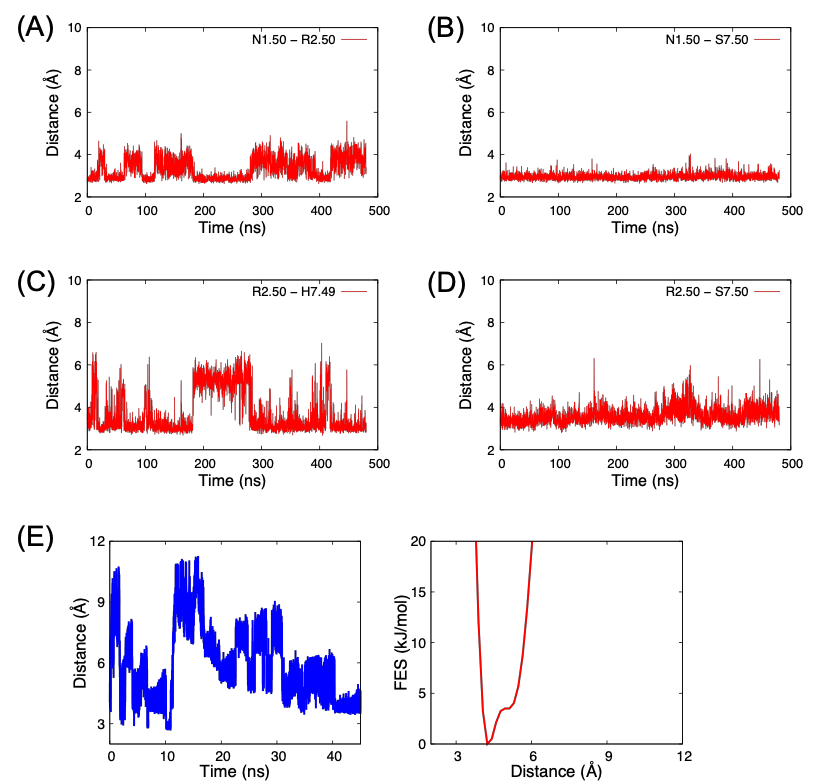


**Fig. S14.** Distances during 480ns MD simulation for (A) N24^1.50^ – R55^2.50^, (B) N24^1.50^ – S277^7.50^, (C) R55^2.50^ – H276^7.49^, and (D) R55^2.50^ – S277^7.50^. (E) Free energy analysis by metaMD for interactions between N24^1.50^ – R55^2.50^.

**Table S7**. The top 10 structures after the BiHelix calculation for predicting TMD of inactive TAS2R4, ordered by neutral interhelical energy (Nih). The 1st and 7th structures (bold) were used for the SuperBiHelix step.

| # | Eta | | | | | | | rankCih | rankNih | rankCNti | Source |
| --- | --- | --- | --- | --- | --- | --- | --- | --- | --- | --- | --- |
|  | H1 | H2 | H3 | H4 | H5 | H6 | H7 |  |  |  |  |
| **1** | **0** | **0** | **0** | **0** | **0** | **-60** | **60** | **18** | **1** | **10** | **5-HT_2C_** |
| 2 | 0 | 0 | 0 | 180 | 0 | -60 | 60 | 9 | 2 | 12 | 5-HT_2C_ |
| 3 | 0 | 0 | 0 | 180 | 0 | 60 | 0 | 3 | 3 | 1 | 5-HT_2C_ |
| 4 | 0 | 0 | 0 | 150 | 0 | -60 | 60 | 22 | 4 | 126 | 5-HT_2C_ |
| 5 | 0 | 0 | 0 | 90 | -90 | 0 | 0 | 30 | 5 | 6 | 5-HT_2C_ |
| 6 | 0 | 0 | 0 | 180 | -60 | 60 | 0 | 4 | 6 | 7 | 5-HT_2C_ |
| **7** | **0** | **0** | **0** | **180** | **0** | **0** | **0** | **13** | **7** | **2** | **5-HT_2C_** |
| 8 | 0 | 0 | 0 | 180 | -90 | 0 | 0 | 1 | 8 | 3 | 5-HT_2C_ |
| 9 | 0 | 0 | 30 | 90 | 180 | 60 | 0 | 31 | 9 | 203 | 5-HT_2C_ |
| 10 | 0 | 0 | 0 | 180 | -30 | 60 | 0 | 5 | 10 | 29 | 5-HT_2C_ |

**Table S8**. The top 10 structures after the SuperBiHelix calculation, which was ordered by total energy (CNti), and the 1st structure (bold) was used for the inactive TAS2R4 structure.

| # | Theta | | | | | | | Phi | | | | | | | Eta | | | | | | | rankCih | rankNih | rankCNti | Source |
| --- | --- | --- | --- | --- | --- | --- | --- | --- | --- | --- | --- | --- | --- | --- | --- | --- | --- | --- | --- | --- | --- | --- | --- | --- | --- |
|  | H1 | H2 | H3 | H4 | H5 | H6 | H7 | H1 | H2 | H3 | H4 | H5 | H6 | H7 | H1 | H2 | H3 | H4 | H5 | H6 | H7 |  |  |  |  |
| **1** | **-10** | **-10** | **0** | **0** | **10** | **-10** | **-10** | **-30** | **15** | **15** | **-30** | **-30** | **-15** | **-30** | **0** | **15** | **0** | **-30** | **0** | **0** | **15** | **58** | **96** | **1** | **7th** |
| 2 | -10 | -10 | 0 | 0 | 10 | -10 | -10 | -30 | 15 | 15 | -30 | -30 | -30 | -30 | 0 | 30 | 0 | -30 | 0 | 0 | 15 | 23 | 48 | 2 | 7th |
| 3 | -10 | -10 | 0 | 0 | 10 | -10 | -10 | -30 | 15 | 15 | -30 | -30 | 0 | -15 | 0 | 15 | 0 | -30 | 0 | 0 | 15 | 64 | 120 | 3 | 7th |
| 4 | -10 | -10 | 0 | 0 | 10 | -10 | -10 | -30 | 15 | 15 | -30 | -30 | -30 | -30 | 0 | 15 | 0 | -30 | 0 | 0 | 15 | 88 | 136 | 4 | 7th |
| 5 | 0 | 0 | 0 | 0 | 10 | -10 | -10 | -15 | 15 | 0 | -30 | -15 | -30 | 0 | 0 | 0 | 0 | -15 | 15 | 0 | 15 | 112 | 98 | 5 | 7th |
| 6 | 0 | 0 | 0 | 0 | 10 | -10 | -10 | -15 | 15 | 15 | 0 | -30 | -15 | -30 | -15 | 0 | 0 | -30 | 0 | 0 | 15 | 101 | 52 | 6 | 7th |
| 7 | 0 | 0 | 0 | 0 | 10 | -10 | -10 | -15 | 15 | 0 | 0 | -30 | -30 | 0 | -15 | 0 | 0 | -30 | 15 | 0 | 15 | 29 | 122 | 7 | 7th |
| 8 | 0 | -10 | -10 | 0 | 0 | -10 | 0 | -30 | -30 | 0 | -15 | -15 | -30 | 30 | 15 | 0 | 30 | 0 | 0 | -15 | 15 | 48 | 186 | 8 | 7th |
| 9 | -10 | 0 | 0 | 0 | 10 | -10 | -10 | 0 | 15 | 15 | -15 | -30 | -15 | -30 | -15 | 15 | 0 | -30 | 15 | 0 | 15 | 13 | 7 | 9 | 7th |
| 10 | -10 | -10 | -10 | 0 | 0 | -10 | -10 | 0 | 15 | 0 | -15 | -15 | 30 | -15 | 0 | 0 | 30 | 0 | 0 | -15 | 15 | 20 | 152 | 10 | 7th |


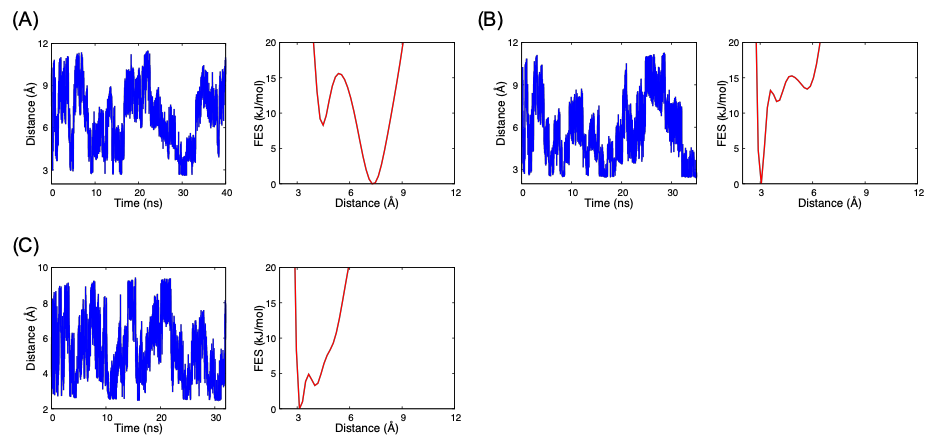


**Fig. S15.** Free energy analysis by metaMD for interactions between (A) N24^1.50^ – R55^2.50^, (B) R55^2.50^ – S95^3.39^, and (C) N24^1.50^ – S277^7.50^, respectively, for the inactive TAS2R4 structure.

**
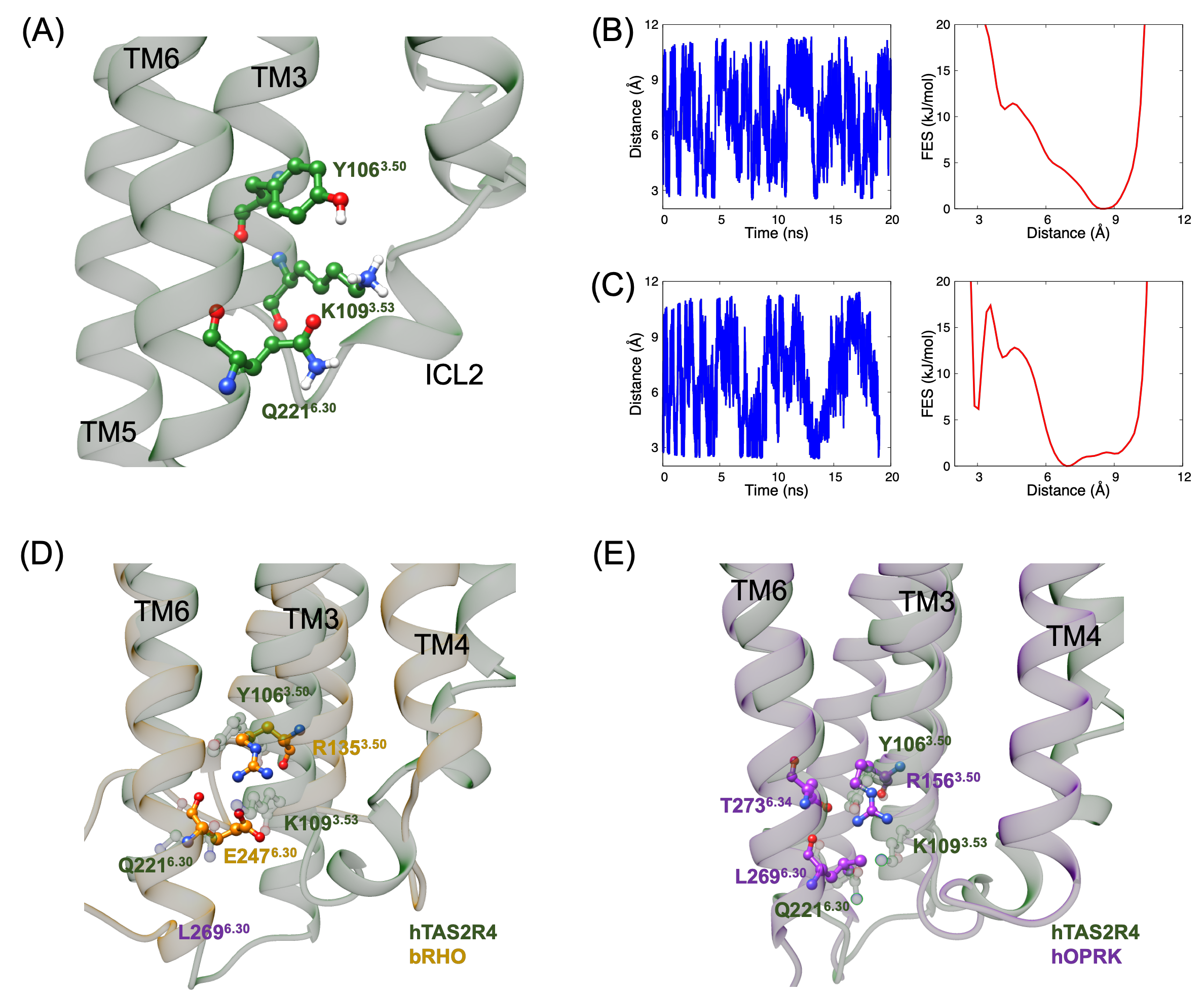
**

**Fig. S16**. (A) Candidate residues for the TM3-6 HB interaction in the inactive structure. Free energy analysis by metaMD for interactions (B) between Y106^3.50^ and Q221^6.30^, and (C) between K109^3.53^ and Q221^6.30^, respectively. There is neither a hydrogen bond or a salt bridge to form an ionic lock. Comparisons of TAS2R4 with (D) bovine rhodopsin (bRHO) and (E) human kappa opioid receptor (hOPRK), where bRHO has a R^3.50^ – E^6.30^ SB and hOPRK has a R^3.50^ – T^6.34^ HB, respectively.

**References**

1. Cvicek V, Goddard WA, 3rd, Abrol R (2016) Structure-based sequence alignment of the transmembrane domains of all human GPCRs: phylogenetic, structural and functional implications. *PLoS Comput Biol* 12(3):e1004805.

2. Pándy-Szekeres G, Munk C, Tsonkov TM, Mordalski S, Harpsøe K, Hauser AS, Bojarski AJ, Gloriam DE (2018) GPCRdb in 2018: adding GPCR structure models and ligands, *Nucleic Acids Res* 46(D1):D440–D446.

3. Wiener A, Shudler M, Levit A, Niv MY (2012) BitterDB: a database of bitter compounds. *Nucleic Acids Res* 40:D413–D419.
